# Increment of Lysosomal Biogenesis by Combined Extracts of Gum Arabic, Parsley, and Corn Silk: A Reparative Mechanism in Mice Renal Cells

**DOI:** 10.1155/2020/8631258

**Published:** 2020-07-11

**Authors:** Aya Helmy, Mohamed El-Shazly, Nesreen Omar, Mohamed Rabeh, Usama Ramadan Abdelmohsen, Reham Tash, Mohammad Alaraby Salem, Ahmed Samir, Ali Elshamy, Abdel Nasser B. Singab

**Affiliations:** ^1^Department of Pharmacognosy, Faculty of Pharmacy, Modern University for Technology and Information (MTI), Cairo 11571, Egypt; ^2^Department of Pharmacognosy, Faculty of Pharmacy, Ain Shams University, Organization of African Unity Street, Abassia, Cairo 11566, Egypt; ^3^Department of Pharmaceutical Biology, Faculty of Pharmacy and Biotechnology, German University in Cairo, Cairo 11835, Egypt; ^4^Department of Biochemistry, Faculty of Pharmacy, Modern University for Technology and Information (MTI), Cairo 11571, Egypt; ^5^Department of Pharmacognosy, Faculty of Pharmacy, Cairo University, Cairo 11562, Egypt; ^6^Department of Pharmacognosy, Faculty of Pharmacy, Minia University, Minia 61519, Egypt; ^7^Department of Pharmacognosy, Faculty of Pharmacy, Deraya University, Universities Zone, New Minia City 61111, Egypt; ^8^Department of Anatomy and Embryology, Faculty of Medicine, Ain Shams University, Abassia, Cairo 11566, Egypt; ^9^Department of Anatomy and Embryology, Faculty of Medicine, King Abdulaziz University, Rabigh 25724, Saudi Arabia; ^10^Department of Pharmaceutical Chemistry, Faculty of Pharmacy, October University of Modern Sciences and Arts (MSA), Giza 12585, Egypt

## Abstract

Gum Arabic (GA), parsley, and corn silk have been traditionally used for renal failure patients worldwide. This study aimed at probing the mechanism of the combined extracts, namely, GA (3 g/kg/day), parsley (1 g/kg/day), and corn silk (200 mg/kg/day), as nephroprotective agents in mice after amikacin (1.2 g/kg) single dose through exploration of their action on G-protein coupled receptors (GPR) 41 and 43 and the ensuing lysosomal biogenesis. Western blotting was employed for renal levels of bcl-2-associated X protein (BAX) and cytosolic cathepsin D; cell death markers, nuclear transcription factor EB (TFEB), and lysosomal associated membrane protein-1 (LAMP-1); and lysosomal biogenesis indicators. Liquid chromatography–mass spectrometry (LC-MS) and docking were also employed. After amikacin treatment, BAX and cathepsin D levels were upregulated while LAMP-1 and nuclear TFEB levels were inhibited. The combined extracts inhibited BAX and cytosolic cathepsin D but upregulated LAMP-1 and nuclear TFEB levels. Docking confirmed GPR modulatory signaling. The combined extracts showed GPR signal modulatory properties that triggered lysosome synthesis and contributed to reversing the adverse effects of amikacin on renal tissues.

## 1. Introduction

Aminoglycoside antibiotics are hydrophilic polar compounds that are prescribed for the treatment of serious Gram-negative and multiresistant infections [1]. The hydrophilic nature of this group of compounds was a vice for the renal wellbeing, resulting in nephrotoxicity, which represented a barrier for the routine utilization of aminoglycosides [2]. In the last decade, protocols for the use of aminoglycoside antibiotics were modified, favoring a single one-off dose to maintain a good renal outcome [3]. Among these antibiotics, amikacin is prescribed in a single one-off dose as a perioperative prophylactic antibiotic to control infections among hospitalized patients [4]. It is also administered to treat gonorrhea. However, the single one-off dose of aminoglycosides also produced negative effects on the urinary system [5]. Amikacin was found to target renal proximal tubular cells provoking acute kidney injury (AKI), which can cause, over a long period of time, chronic kidney disease (CKD) [6]. Aminoglycosides induce cytotoxicity to the cells of the urinary system by promoting oxidative and physical stresses as well as impairing lysosome functions and interfering with autophagy. Basically, autophagy is a restorative process supporting cellular survival in times of stress as it degrades misfolded proteins and damaged organelles to generate free building units for energy compensation [7]. In this way, the accumulation of toxic aminoglycosides in the epithelial cells of the proximal renal tubules drives renal cell death that cannot be embraced because of the dysfunctional autophagy. Therefore, it is vital to reverse acute renal toxicity by the coadministration of therapeutic agents that are able to infiltrate into injured renal cells and manipulate cellular machinery to restore autophagy.

The role of herbal medicine such as gum Arabic, parsley, and corn silk in improving renal parameters is well documented [8]. The single use of any of these herbs for renal disorders is practiced in folk medicine [9]. However, the increasing frequency of prescribing mixtures of these herbs by practitioners of natural medicine calls for careful examination [10]. The use of these herbs results in a general improvement in the urinary system wellbeing through the reduction in blood urea nitrogen (BUN) and creatinine concentration [11], but the precise signaling cascades they elicit to repair renal cells remain obscure. Therefore, the benefits of combining the extracts of gum Arabic, parsley, and corn silk on the urinary system following a single one-off dose of amikacin are worth investigation.

The aim of the current study was to revisit the mechanism of renal damage by a single one-off dose of amikacin. Additionally, we investigated the potential of GA derived SCFAs for lysosomal biogenesis and of parsley and corn silk for preserving lysosomes to impart renal cell repair in the light of their respective constituent compounds.

## 2. Materials and Methods

### 2.1. Animals

All animal procedures and care were conducted according to the general guidelines of the Research Ethics Committee of the Faculty of Medicine, Ain Shams University, which conformed to the guiding principles of the International Council on Harmonization and the Islamic Organization for Medical Sciences, the United States Office for Human Research Protections, and the United States Code of Federal Regulations and operated under Federal Wide Assurance No. FWA00006444. Thirty male Albino mice (25–30 g) 2–3-month-old were selected from the Laboratory Animals Research Center in the Faculty of Medicine, Ain Shams University. The mice were maintained under controlled temperature and 12-hour light/12-hour dark conditions for one week before starting the experiments. They were allowed to feed on standard laboratory chow and tap water ad libitum.

### 2.2. Drugs and Chemicals

Parsley (*Petroselinum crispum* (Mill.) Fuss) herb, corn silk (*Stigma maydis*) from *Zea mays* L., and gum Arabic (obtained from *Acacia senegal* (L.) Willd. Trees) were identified by Dr. Nada Mostafa (Pharmacognosy Department, Faculty of Pharmacy, Ain Shams University). A voucher sample was kept at Pharmacognosy Department, Faculty of Pharmacy, Ain Shams University, with the following numbers: parsley PHG-P-PC 198, corn silk PHG-P-ZM 197, and gum Arabic PHG-P-AS 199. Parsley herb and corn silk were washed with tap water and then all impurities were removed by distilled water. They were then dried under shade for several days at room temperature to remove any moisture. GA was sieved to remove any foreign matter. The dried plant parts and the sieved GA were then ground using an electric blender to obtain a fine powder that was stored in amber bottles.

### 2.3. Preparation of Aqueous Extracts

The aqueous extracts were prepared by adding 200 g of air-dried plants parts (parsley herb or corn silk) to 1 L of distilled water followed by boiling for 30 min. For gum Arabic, 500 g was extracted by adding 1 L of distilled water followed by boiling for 30 min. The extracts were then filtered, and the filtrates were evaporated using a rotary evaporator under reduced pressure to dryness, then lyophilized, and weighed. The extraction yields were 2.66, 3, and 185 g for parsley, corn silk, and gum Arabic, respectively. A combined extract was formulated of the three aqueous extracts by mixing a specific weighed amount of each. All the lyophilized aqueous extracts with the combined extract were then dissolved in distilled water prior to administration to the mice.

### 2.4. Experimental Design

The planned study duration was four weeks. Following an initial injection of amikacin (1.2 g/kg i.p. as a single dose) randomly chosen male mice were divided into the control (*n* = 5) and renal impairment (*n* = 25) groups. The control group received distilled water by gavage for 28 days. The renal impairment group was further subdivided into 5 subgroups (5 animals each). The first subgroup received distilled water by gavage for 28 days. The other subgroups were given lyophilized aqueous extract of gum Arabic (3 g/kg/day for 28 days by gavage), lyophilized aqueous extract of parsley herb (1 g/kg/day for 28 days by gavage), and lyophilized aqueous extract of corn silk (200 mg/kg for 28 days by gavage) and the last group were given a combined extract formulated from the three previously mentioned lyophilized aqueous extract at the same dosing levels for 28 days. The administration of drugs was initiated one day after amikacin administration.

### 2.5. Blood Collection and Tissue Processing

After 28 days of amikacin administration, blood samples were collected from the retro-orbital plexus and allowed to clot. Plasma was separated by centrifugation at 3000 ×g for 15 min and used for the assessment of urea, creatinine, sodium, and reduced glutathione (GSH). Mice were then sacrificed by rapid decapitation; both kidneys were rapidly dissected and immersed in cooled (2–8°C) 0.9% NaCl solution. One kidney was fixed in 10% formalin for histopathological study. The second kidney was immediately frozen on dry ice and stored at −80°C for later analysis of malondialdehyde (MDA) via a fluorometric assay and protein expression with Western blot.

### 2.6. Biochemical Investigations

Urea, creatinine, sodium, and reduced GSH levels were determined in plasma samples using enzymatic and colorimetric methods using commercial kits, and the data were expressed as mg/dL. MDA levels were determined by the fluorometric method described earlier [12] based on thiobarbituric acid (TBA) reactivity. In brief, 50 *μ*L of the homogenate or an adequate volume of MDA working standard solution was introduced into 10 mL glass tubes containing 1 mL of distilled water. After adding 1 mL of the solution containing 29 mmol/L TBA in acetic acid (pH of the reaction mixture, 2.4–2.6) and mixing, the samples were placed in a water bath and heated for 1 h at 95–100°C. After the samples were cooled, 25 *μ*L of 5 mol/L HCl was added (final pH 1.6–1.7), and the reaction mixture was extracted by agitation for 5 min with 3.5 mL of n-butanol. We separated the n-butanol phase by centrifugation at 1500 ×g for 10 min and the fluorescence of the n-butanol extract was measured with a fluorometer at wavelengths of 525 nm for excitation and 547 nm for emission.

### 2.7. Tissue Preparation for Western Blot Analysis

Renal cortical tissue was chopped and homogenized on ice in mammalian cell lytic buffer with a protease inhibitor cocktail. Each cellular component, whole-cell lysate, membrane, and cytosolic fractions were prepared from renal cortical slices using differential centrifugation as previously described [13]. Briefly, the homogenate was centrifuged at 5000 ×g for 10 min at 4°C, the supernatant was designated as whole-cell lysate, and then the supernatant was further centrifuged at 100000 ×g for 2 h at 4°C to obtain membrane (pellet) and cytosolic (supernatant) fractions. The 5000 g pellet was resuspended and centrifuged at 10000 ×g at 4°C for 10 min. The supernatant fraction from the spin was designated as the nuclear fraction. All the fractions collected were stored at −80°C until use.

### 2.8. Western Blot

After centrifugation, the cytoplasmic fraction was used for the determination of cytosolic cathepsin D and the nuclear fraction was used for evaluating the transcription factor EB (TFEB) in the nucleus. The whole-cell lysate was assigned to verify lysosome-associated membrane protein-1 (LAMP-1) and Bcl-2-associated X protein (BAX). Tissue protein was then extracted using TRIzol reagent, and protein concentrations were estimated by the Bradford method. Equal amounts of protein per lane were separated with 10% SDS polyacrylamide gel electrophoresis and electrophoretically transferred to polyvinylidene difluoride (PVDF) membranes. Membranes were then incubated at room temperature for 2 h with blocking solution comprised of 5% nonfat dried milk in 10 mM Tris-Cl, pH 7.5, 100 mM NaCl, and 0.1% Tween 20. Membranes were incubated overnight at 4°C with the indicated primary antibodies against beta-actin, cathepsin D, LAMP-1, TFEB (1 : 200, Santa Cruz Biotechnology, Inc.), lamin B1 (1 : 1000, Santa Cruz Biotechnology, Inc.), and BAX (1 : 500, Santa Cruz Biotechnology), and then incubated with a mouse anti-rabbit secondary monoclonal antibody conjugated to horseradish peroxidase at room temperature for 2 h. After each incubation, the membranes were washed four times with 10 mM Tris-Cl, pH 7.5, 100 mM NaCl, and 0.1% Tween 20 at room temperature. Chemiluminescence detection was performed with the Amersham detection kit according to manufacturer's protocols. The amount of the studied protein was quantified by densitometric analysis using Bio-Rad software, USA. Results were expressed as arbitrary units after normalization for *β*-actin protein expression.

### 2.9. Histopathological Examination

Kidney tissues were fixed in 10% formalin overnight and embedded in paraffin. Serial sections of 4 *μ*m thick were stained with hematoxylin and eosin for light microscopic histological examination. In all renal samples, at least three kidney sections, after the fifth cut, were chosen to evaluate glomerular and tubular cells throughout the entire renal cortex using a digital video camera mounted on a light microscope (CX31, OLYMPUS, Japan).

### 2.10. Metabolomic Profiling of Petroselinum crispum and Stigma maydis and a Combination of the Three Crude Extracts

Metabolomic profiling was performed on crude extracts of *P. crispum* and *S. maydis* and a combination of the three extracts to deliver general qualitative and quantitative profiles of metabolites that may be involved in the activity of the extracts [14, 15]. Dereplication refers to the rapid identification of known secondary metabolites and their quantification in crude unfractionated extracts [16, 17]. LC-MS measurement was done on an Acquity Liquid Chromatography (LC) system coupled to a Synapt G2 HDMS quadrupole time-of-flight hybrid mass spectrometer (Waters, Milford, USA). Chromatographic separation was carried out on a BEH C18 column (2.1 × 100 mm, 1.7 *μ*m particle size; Waters, Milford, USA) with a guard column (2.1 × 5 mm, 1.7 *μ*m particle size) and a linear binary solvent gradient of 0%–100% eluent B over 6 min at a flow rate of 0.3 mL·min^−1^, using 0.1% formic acid in water (v/v) as solvent A and acetonitrile as solvent B. The injection volume was 2 *μ*L and the column temperature was 40°C. To convert the raw data into separate positive and negative ionization files, MS converter software was used. The files were then imported to the data mining software MZmine 2.10 for peak picking, deconvolution, deisotoping, alignment, and formula prediction. The database used for the identification of compounds was the Dictionary of Natural Products (DNP) 2015.

### 2.11. Docking

As the case with many GPCRs, the crystal structures of GPR41 (FFAR3) and GPR43 (FFAR2) are not available. Therefore, homology models were sought and downloaded from the SWISS-MODEL repository with IDs O15552 and O14843, respectively. For FFAR2, the template protein was the human protease-activated receptor-2 (PAR2) with crystal structure PDB ID 5nj6. For FFAR3, the template protein was the lysozyme, proteinase-activated receptor-2 with crystal structure PDB ID 5ndd.1.A. Sequence identities are 25.09% and 28.57%, respectively. The active sites were determined via homology to another GPCR which had a cocrystallized ligand, namely, human protease-activated receptor 1 (PAR1) with PDB ID 3vw7.

In all dockings, a grid box of dimensions 50 grid points and spacing 0.375 was centered on the given ligand. Docking was performed via Autodock4 implementing 100 steps of the genetic algorithm while keeping all the default settings provided by Autodock Tools [18]. Visualization was done using Discovery Studio.

## 3. Results

### 3.1. Effects of Gum Arabic, Parsley, and Corn Silk Aqueous Extracts and the Combined Extract on Serum Renal Parameters

**Figure 1:**
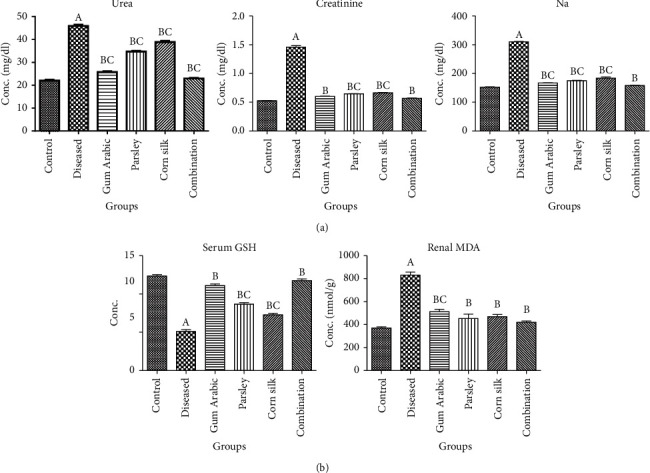
Effect of amikacin, gum Arabic, parsley, and corn silk and combination of them on serum urea, creatinine, and sodium (a), in addition to serum reduced glutathione and renal malondialdhyde (b). Data are presented as means ± SD. ^A^Significant (*P* < 0.05) versus control; ^B^Significant (*P* < 0.05) amikacin group; ^C^Significance of single treatment (*P* < 0.05) versus combination group; one-way ANOVA followed by Bonferroni-corrected post hoc tests.

A single one-off dose of amikacin induced a significant increase (*P* < 0.05) in serum urea, creatinine, and sodium in the nontreated group compared with the normal control group (Figure 1(a)). Administration of gum Arabic, parsley, and corn silk aqueous extracts and the combined extract for 28 days in amikacin treated groups improved renal functions, as they significantly decreased urea, creatinine, and sodium (*P* < 0.05).

### 3.2. Effects of Gum Arabic, Parsley, and Corn Silk Aqueous Extracts and the Combined Extract on Oxidative Stress Markers in Serum and Renal Tissues

Gum Arabic, parsley, and corn silk aqueous extracts and their combination yielded significantly higher levels of reduced GSH in serum and lower levels (*P* < 0.05) of MDA in renal tissues compared to the amikacin group (Figure 1(b)).

### 3.3. Induction of Renal Cell Death by Amikacin and the Reversing of This Effect by Gum Arabic, Parsley, and Corn Silk Aqueous Extracts and the Combined Extract

**Figure 2:**
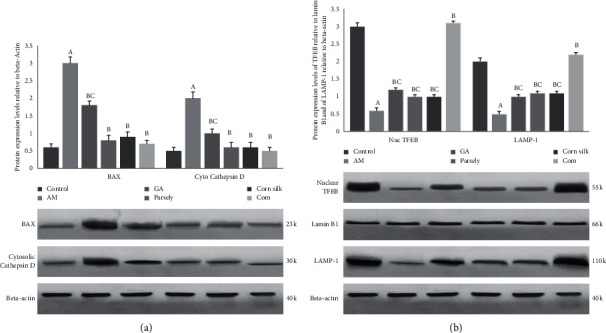
Effect of amikacin, gum Arabic, parsley, and corn silk and combination of them on protein expression of BAX, LAMP-1, cytoplasmic cathepsin D, and nuclear TFEB in renal tubular cells by Western blot analysis. (a) Representative Western blot for BAX and cytoplasmic cathepsin (D). In densitometric quantification of Western blot, the bars represent the ratio ± SD versus beta-actin. (b) Representative Western blot for LAMP-1 and nuclear TFEB. In densitometric quantification of Western blot, the bars represent the ratio ± SD versus beta-actin for LAMP-1 and versus lamin B1 for nuclear TFEB. ^A^Significant (*P* < 0.05) versus control. ^B^Significant (*P* < 0.05) versus amikacin group. ^C^Significance of single treatment (*P* < 0.05) versus combination group; one-way ANOVA followed by Bonferroni-corrected post hoc tests.

In the present study, BAX, an apoptotic protein, and cytosolic cathepsin D, an indicator of lysosomal membrane rupture, were determined. As shown in Figure 2(a), the amikacin group showed an increase in the expression of BAX (5-fold) and cathepsin D (4-fold) compared with the control group (*P* < 0.05). The administration of gum Arabic, parsley, and corn silk aqueous extracts to the amikacin treated groups resulted in downregulating BAX expression by 1.6-, 3.7-, and 3.3-fold in addition to the cytosolic expression of cathepsin D by 2-, 3.3- and 3.2-fold, respectively, compared with the amikacin only treated group (*P* < 0.05). When the combination of the three aqueous extracts was administered to an amikacin treated group for 28 days, the protein expression of both BAX and cathepsin D was restored back to the level of the control group (*P* < 0.05).

### 3.4. The Decline of Lysosomal Biogenesis by Amikacin and Readdressing the Harm by Gum Arabic, Parsley, and Corn Silk Aqueous Extracts and the Combined Extract

In the present study, the protein expression of TFEB in the nuclear fraction of renal cells together with the expression of LAMP-1 was inhibited (5- and 4-fold, resp., *P* < 0.05) after a single one-off dose of amikacin. The administration of gum Arabic, parsley, and corn silk aqueous extracts for 28 days after amikacin single dose upregulated the expression of nuclear TFEB. Localization of TFEB in the nucleus indicates active transcription of the lysosomal genes. This can be viewed through the active expression of LAMP-1, an indication of lysosome abundance. In this study LAMP-1 was actively expressed after gum Arabic, parsley, and corn silk administration by 2-, 1.6- and 1.7-fold, respectively, compared with the amikacin group. Nuclear TFEB and LAMP-1 expression in the combination treatment group were not significantly different from those in the control group (Figure 2(b)).

### 3.5. Renal Histology

**Figure 3:**
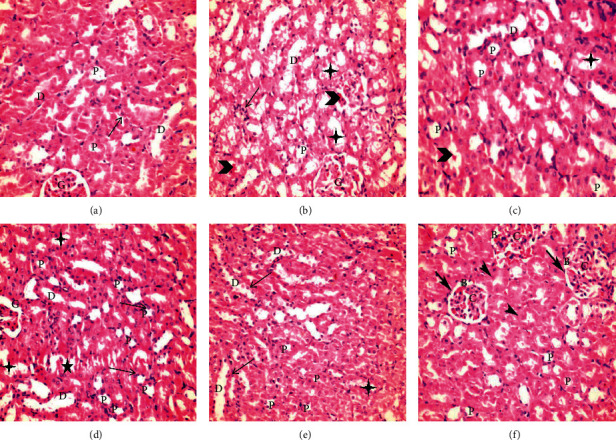
Photomicrographs of histological sections of mice renal cortex by hematoxylin and eosin (HE х400). *Normal control* (a): normal renal cortex can be seen with normal glomerular tuft (G), normal proximal convoluted tubules (P) with vesicular nuclei (arrow), normal distal convoluted tubules with vesicular nuclei (D). *Amikacin group* (b) shows atrophied vacuolated cytoplasmic epithelium of the distal convoluted tubules with pyknotic nuclei (D), atrophied vacuolated cytoplasmic epithelium of the proximal convoluted tubules with pyknotic nuclei (P), interstitial proliferated tissue (arrow), lobulation of the glomerular tuft (G), abundance of dense acidophilic hyaline casts (arrowhead), and cellular debris in tubular lumina (stars). *Gum Arabic group* (c) shows the proximal convoluted tubules (P) that are lined with high cuboidal cells with rounded vesicular basal nuclei (arrow) and deeply acidophilic cytoplasm, the distal convoluted tubules (D) have wider lumina and are lined with cubical cells with rounded vesicular central nuclei and paler acidophilic cytoplasm, and there are still few cellular debris (stars) and hyaline casts (arrowhead). *Parsley group* (d) shows normal glomerular tuft (G), some normal proximal convoluted tubules (P) with vesicular nuclei (arrow), and normal distal convoluted tubules (D); some of them show cellular vesicular debris (stars). *Corn silk group* (e) shows normal proximal convoluted tubules (P) with vesicular nuclei (arrow) and narrow lumen, normal distal convoluted tubules (D), with abundant vesicular nuclei (arrow), and few cellular debris. *Combination group* (f): the renal cortex shows malpighian renal corpuscle (arrow) containing glomerulus (G) and nondilated Bowman's space (B), normal proximal convoluted tubules (P) with vesicular nuclei (arrowhead) and narrow lumina, normal distal convoluted tubules (D), and no cellular debris or hyaline casts present. A reclaim of renal architecture is obvious.

Renal glomeruli and proximal tubular cells were examined in the mice fed for 28 days after the administration of a single one-off dose of amikacin and in mice treated with gum Arabic, parsley, and corn silk aqueous extracts and the combined extract following amikacin administration. The amikacin group showed marked tubular degeneration and glomerular atrophy, while single treatment groups demonstrated intact tubular cells. A varying degree of distorted architecture in the form of cellular debris or hyaline casts in the tubular lumen was observed. The combined extract group showed intact glomeruli, the proximal convoluted tubules PCTs were lined with cuboidal epithelium, and the distal convoluted tubules DCTs were lined with columnar epithelium with no signs of cellular degeneration (Figure 3).

### 3.6. Metabolomic Profiling Results

Identification of fifteen compounds was achieved using the Dictionary of Natural Products (DNP). These compounds belong to various classes of active constituents including alkaloids, flavonoids, and phytosterols (Table 1).

### 3.7. Docking Results

**Figure 4:**
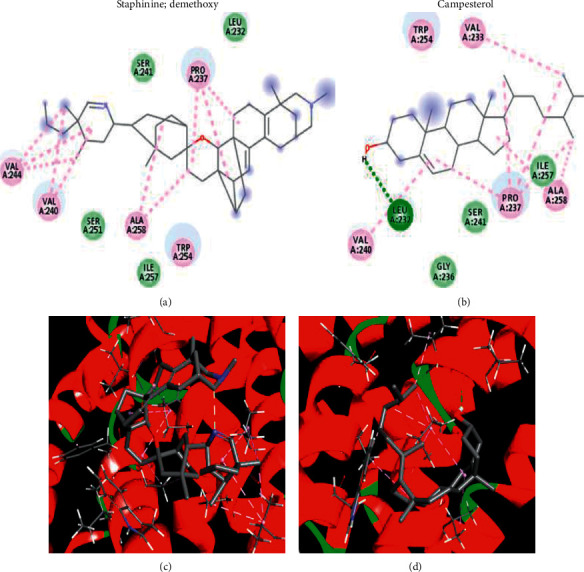
2D interactions (top) and 3D plots (bottom) of top-scoring ligands in the predicted active site of the homology model of GPR43 (FFAR2). Purple and light green refer to hydrophobic and van der Waals interactions, respectively. For campesterol (right), solid green refers to H-bond interaction with Leu232.

**Figure 5:**
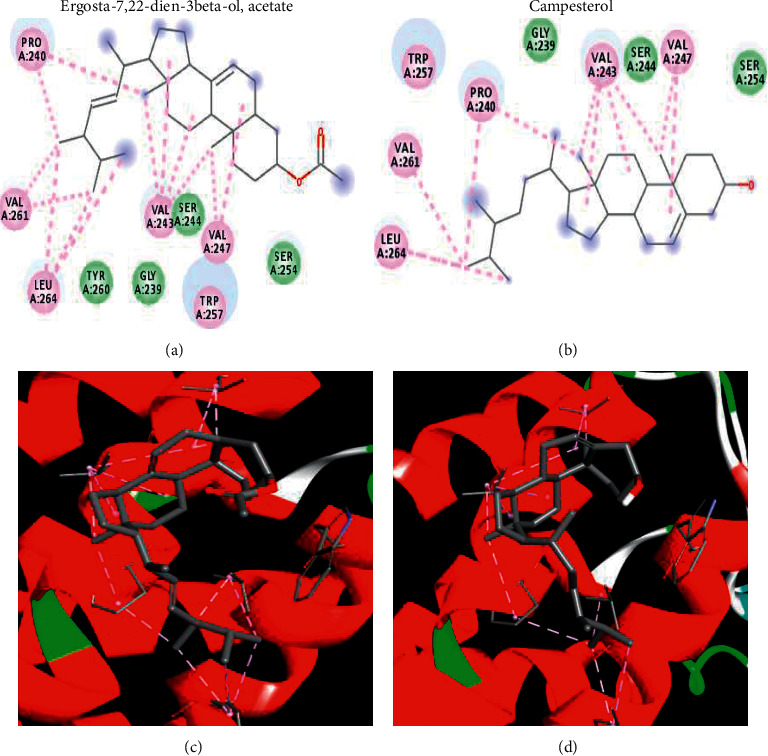
2D interactions (top) and 3D plots (bottom) of top-scoring ligands in the predicted active site of the homology model of GPR41 (FFAR3). Purple and light green refer to hydrophobic and van der Waals interactions, respectively.

Docking results showed that eleven compounds (out of fifteen) that were identified through LC-MS had high docking scores against both FFAR2 and FFAR3 (Table 2). The interactions for the top-scoring ligands are dominated by hydrophobic interactions with FFAR2. As illustrated in Figures 4 and 5, the predicted pocket of FFAR2 has many hydrophobic amino acids, like valine and alanine that adhered to the hydrophobic nucleus of the top-scoring ligands. Only one hydrogen bond was observed with campesterol and the carbonyl linked to the alpha carbon of Leu 232. The same argument held true for docking in the homologous active site of FFAR3. The pocket has several hydrophobic amino acids that form hydrophobic and van der Waals interactions with the hydrophobic ligands. The current docking study presents an explanation for the observed activity on the molecular level.

## 4. Discussion

The clinical application of aminoglycosides faced a lot of setbacks due to their severe renal cytotoxicity which targets the proximal tubular cells through multipronged mechanisms [36]. Aminoglycosides bind to cell membrane phosphoinositides, promoting the generation of reactive oxygen species (ROS), bind to mitochondrial ribosomes, disrupt protein synthesis, and cause mitochondrial damage [37]. Moreover, aminoglycosides occupy lysosomes and increase the lysosomal membrane permeabilization (LMP), disarming the cell of the autophagy survival outlet [7]. An outcome of atrophied and nonfunctional nephrons is anticipated but veiled by adaptive nephrons hypertrophy which undergoes hyperfiltration to preserve the kidney functions regardless of the elicited damage [38]. Therefore, the insidious clinical presentations of aminoglycosides induced acute renal failures like nonoliguric, slow onset and low daily rise of plasma creatinine, warranted our attention [39].

The prebiotic gum Arabic or gum acacia (GA) is a dietary soluble fibrous and complex heteropolysaccharide obtained from *Acacia senegal* (L.) Willd. Trees [40]. In chronic renal failure, GA was found to act as “enterosorbent,” lowering the circulating levels of urea and creatinine which ultimately inhibit inflammation and oxidative stress [41]. Consequently, GA can slow down the progression of kidney damage; however, the role of GA in repairing damaged kidney tissues needs consolidation. Given that GA is indigestible but is fermented in the large intestine by microorganisms, GA produces short chain fatty acids (SCFAs), specifically propionic acid [42]. SCFAs are ligands for G-protein coupled receptors (GPR41, GPR43, GPR109 A, and olfactory receptor 78) or can act as epigenetic regulators (HDAC inhibitors) [43].

Parsley herb and corn silk are herbal products that contain a varied amount of polyphenolics, alkaloids, and flavonoids which are known to possess antioxidant properties [44, 45]. One of the results of the colossal oxidative stress, which is a consequence of the administration of aminoglycosides, is lipid peroxidation in lysosomal membranes [46]. However, it is questionable whether the extent of aminoglycoside oxidation will cause destabilization of the lysosomal membrane, which barricades autophagic response and may trigger apoptotic pathways. Hence, this study was set out to analyze the impact of parsley and corn silk extracts on the integrity of the lysosomal membrane as measured via lysosome-associated membrane protein-1 (LAMP-1), which is the main component of the lysosomal membrane proteins. Identifying the urinary system protective effect without knowing the composition of the herbal mixture does not provide a clear picture of the potential applications of the herbal extracts. So, we identified the components of parsley and corn silk using a reliable, robust, and selective LC-MS (dereplication) protocol. The possible association of the identified secondary metabolites with GPR was further challenged using a docking experiment.

In the present study, the administration of amikacin to Albino mice in a single dose caused severe renal damage evident by significantly higher concentrations of urea, creatinine, and sodium than the control group. There was marked oxidative stress as indicated by the decreased concentration of reduced GSH in serum and increased MDA in renal tissues relative to the control group. Renal cells in the amikacin group showed significantly high levels of both the proapoptotic protein BAX and the cytosolic cathepsin D, a lysosomal hydrolase that marks lysosomal membrane permeabilization (LMP). Previous reports demonstrated that the lysosomal cathepsins disseminate BAX apoptotic signals [47].

Our current findings are conceivable because cathepsin D and BAX are located at the nexus of the cell death amplification loop. BAX was reported to act as a pore-forming protein through the lysosomal membrane, thereby liberating lysosomal cathepsin D to the cytosol [48].

In the amikacin group, the expression of LAMP-1 in the whole-cell lysate and that of TFEB in the nucleus were downregulated. TFEB is a transcription factor responsible for increasing the number of lysosomes, and its capture in cytosol impairs this function [49]. This lysosomal transcription failure appears as downregulated LAMP-1 and it exacerbates amikacin cytotoxicity. This effect might be partially due to the binding of cationic antibiotic to the anionic phosphoinositides [37] which interrupts PLC enzymatic action on PIP2 in response to normal GPR signaling.

In the current study, gum Arabic, parsley herb, and corn silk renowned for GPR signaling or antioxidant properties were applied to investigate reshaping of the renal cell via enriching the lysosomal community with new members or creating an antioxidant environment for lysosomes to hold up.

The administration of parsley or corn silk resulted in an improvement in the renal parameters in plasma. An elevation in serum GSH and a decrease in renal MDA levels were noted. Parsley and corn silk intake suppressed the level of BAX, an apoptotic protein. This finding reflects the high content of flavonoids, alkaloids, and phenolic compounds in both parsley and corn silk which endowed it with free radical scavenging activity, a guaranteed suppressor of apoptosis [44, 45]. Parsley and corn silk exhibited a remarkable stabilizing effect on the lysosomal membrane as they decreased the cytoplasmic level of cathepsin D while they increased the protein levels of LAMP-1 in comparison with the amikacin group. Our results confirmed the previously reported association between apoptosis and lysosome rupture [48]. To the best of our knowledge, the effect of parsley and corn silk on GPR signaling has not been previously reported. Both showed higher protein expression of nuclear TFEB, an indication for active lysosomal synthesis. This finding may be in tandem with reinstating GPR signaling as a part of renal cell homeostasis.

GA is well known to trump other types of fibers in the prebiotic properties owing to the avalanche of SCFAs production [50]. Binding of SCFAs to GPR41 (FFAR3) and GPR43 (FFAR2) activates the Gq subclass which stimulates phospholipase C (PLC) [43]. PLC is at the crux of cell proliferation, differentiation, and survival through their function as phosphodiesterases lipase for converting phosphatidylinositol bisphosphate (PIP2) into inositol triphosphate (IP3) and diacylglycerol (DAG). Diacylglycerol activates protein kinase C (PKC), which then activates many transcription factors [51]. PKC has been found to inactivate GSK3, leading to reduced phosphorylation, nuclear translocation, and activation of TFEB, while PKC activates JNK and p38 MAPK, which phosphorylate ZKSCAN3, leading to its inactivation by translocation out of the nucleus [49]. SCFAs emanating from resident microbes promote autophagy and therefore the survival of colon cells [52]. SCFAs suppressed inflammation and apoptosis in AKI while activating the autophagy gene ATG7, providing evidence for the gut-kidney axis [53]. Literature reported that acetate induced lysosomal biogenesis by inhibiting the histone deacetylase action which could be one more mechanism by which SCFAs are an effective intervention [54]. Here, GA group showed an elevated level of the nuclear TFEB protein expression. This compartmentalization in the nucleus is evidence for active lysosomal gene transcription and autophagy [49]. Also, the decreased levels of cytoplasmic cathepsin D, the proapoptotic protein BAX, and the increased LAMP-1, denote intact lysosomes and inactive cell death. In the GA group, oxidative stress had been extinguished, as displayed by higher levels of GSH and lower levels of MDA. This finding can be viewed as part of “GPR induced abundance of more lysosomes hypothesis” which implies more of the intralysosomal heat shock protein 70 (HSP70). HSP70 has been suggested to tackle cellular stress, so keeping the lysosomal membrane from disruption [55].

We examined the effect of administering the combined extract comprised of GA, parsley, and corn silk to mice after they were subjected to amikacin. The combination group showed a steep reduction in the expression of cathepsin D and BAX. The combined extracts resulted in boosting the expression of LAMP-1 in addition to TFEB in the nucleus. These results warranted us to further identify the specific compounds in parsley and corn silk and the combination of the three extracts responsible for the activity using LC-MS. Later, verification of the possible association of the identified compounds with GPR signaling was done through the aid of docking techniques. Interestingly, a number of compounds in both parsley and corn silk were docked in the allosteric binding sites of GPR 41 and 43. Recently, reports indicated that the binding of compounds in the allosteric pockets of these GPR exhibited binding cooperativity, enhanced the binding of the SCFAs in their orthosteric pocket, and thus increased the GPR functioning [56]. This finding helped us to explain the outstanding renoprotective effect of the combined extract formulated of parsley and corn silk and GA aqueous extracts in comparison to the single extract effect. However, that part of the study is qualitative in nature and remains to be strengthened in the future by elucidating the crystal structures of FFAR2 and FFAR3.

## 5. Conclusion

Our work suggested that GA derived SCFAs act as mediators of intracellular GPR survival signals. In addition, our results shifted the traditional perspective of parsley and corn silk from being viewed as simple antioxidants to probable positive allosteric modulators for GPR 41 and 43. This potentiation of the action of the receptor promises that a combination of parsley and corn silk with GA would reap a great benefit. These findings present the combined extract of GA, parsley, and corn silk as an eminent formula to abrogate aminoglycoside nephrotoxicity via biogenesis of lysosomes as well as keeping the lysosome integrity, with the net result of providing functional autophagy that confers resistance to apoptotic cell death.

## Figures and Tables

**Table 1:** Dereplication of the metabolomics of the crude extracts of *Petroselinum crispum* and *Stigma maydis* and the combined extract formulated from GA, *P. crispum*, and *S. maydis* aqueous extracts assembled according to their molecular weight.

*m*/*z*	Rt. (min.)	M. wt.	Name	Source	Molecular formula	References
166.087	2	165.0794853	Hordenine	Corn silk	C_10_H_15_NO	[19]
271.061	3.7	270.0533351	Imperatorin	Parsley	C_16_H_14_O_4_	[20]
295.227	4.8	294.2200031	13-Hydroxy-10-oxo-11-octadecenoic acid; (±)-(E)-form, lactone	Corn silk	C_18_H_30_O_3_	[21]
301.071	3.7	300.0632686	Chrysoeriol	Parsley	C_16_H_12_O_6_	[22, 23]
313.167	4.6	314.1744034	Dihydroxy-dimethoxyflavone	Parsley	C_17_H_14_O_6_	[24]
				Corn silk		
				Combination		
374.269	5.1	373.2614917	Caldaphnidine O; 6-hydroxy	Corn silk	C_23_H_35_NO_3_	[25]
401.316	3.4	400.3083667	Campesterol	Corn silk	C_28_H_48_O	[26, 27]
426.264	6	425.2563037	Lythranidine	Corn silk	C_26_H_35_NO_4_	[28]
441.321	6.4	440.31431217	Ergosta-7,22-dien-3-*β*-ol, acetate	Corn silk	C_30_H_48_O_2_	[26]
443.151	1.5	442.1431217	Epicatechin-3-O-gallate	Parsley	C_22_H_18_O_10_	[29, 30]
				Corn silk		
				Combination		
485.348	6.4	484.340322	Digalloyl glucose	Corn silk	C_20_H_20_O_14_	[31]
579.203	9.6	578.1963653	Apigenin-7-O- neohesperidoside	Parsley Combination	C_27_H_30_O_14_	[32]
579.205	8.6	578.1988849	2″-O-*α*-L-rhamnosyl-6-C-quinovosyl-luteolin	Corn silk Combination	C_27_H_30_O_14_	[33]
591.43	6.3	590.4231738	Staphinine; demethoxy	Parsley	C_41_H_54_N_2_O	[34]
				Corn silk		
				Combination		
676.464	6.6	675.4567127	L-Arabinoside	Corn silk	C_38_H_61_NO_9_	[35]

^*∗*^Rt; retention time (min.), M.wt.; molecular weight.

**Table 2:** The scores (in Kcal/mol) of top-scoring 11 compounds identified from parsley and corn silk as docked in the active sites of GPR43 (FFAR2) and GPR41 (FFAR3) homology models.

Ligand	FFAR2	FFAR3
Staphinine; demethoxy	−7.61	−8.93
Campesterol^*∗*^	−7.57	−9.46
Ergosta-7,22-dien-3beta-ol, acetate^*∗*^	−6.84	−10.27
Epicatechin-3-O-gallate	−6.58	−6.72
Imperatorin^*∗*^	−6.40	−9.03
13-Hydroxy-10-oxo-11-octadecenoic acid; (±)-(E)-form, lactone	−6.20	−8.41
Lythranidine	−6.14	−7.76
2*″-*O-*α*-L-rhamnosyl-6-C-quinovosyl-luteolin	−5.99	−6.36
7,4′-Dihydroxy-3,5-dimethoxyflavone	−5.69	−6.99
L-Arabinoside	−5.69	−8.41
Chrysoeriol	−5.44	−7.24

^*∗*^Top-scoring 3 compounds for FFAR3. The scores are sorted according to FFAR2.

## Data Availability

The data used to support the findings of this study are available from the corresponding author upon request.

## Supplementary Materials

Supplementary Materials Base peak plot.
