# Supplementary figures and images for: Increment of Lysosomal Biogenesis by Combined Extracts of Gum Arabic, Parsley, and Corn Silk: A Reparative Mechanism in Mice Renal Cells
Source: Evid Based Complement Alternat Med. 2020 Jul 11;2020:8631258. doi: 10.1155/2020/8631258 (PMC7369655; doi:10.1155/2020/8631258)

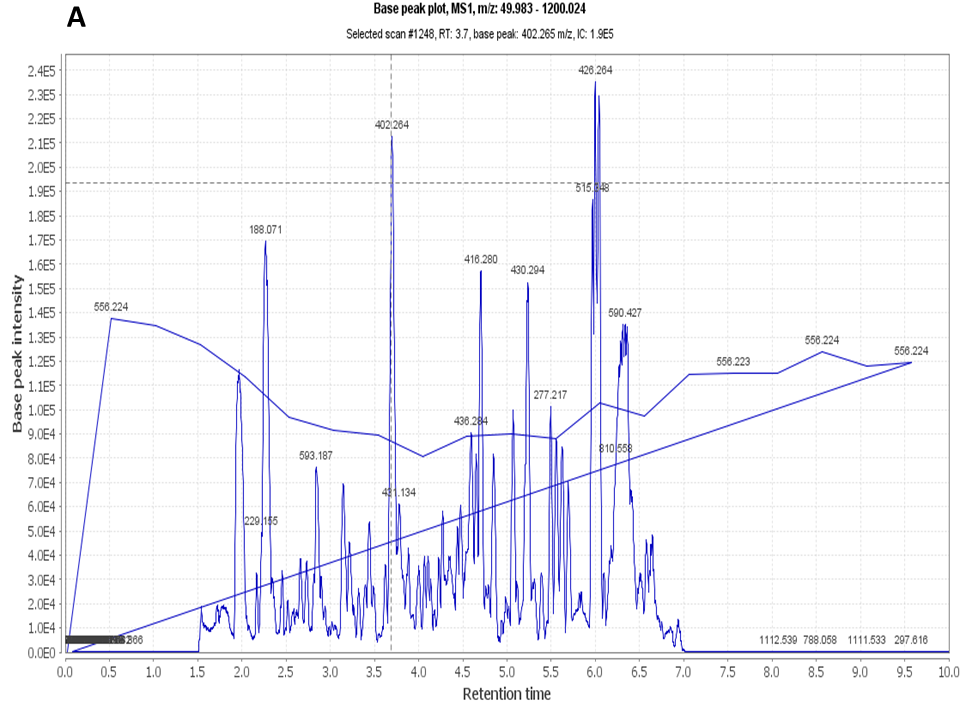

Supplement: Supplementary Materials — Base peak plot. [file 8631258.f1.tif]
